# Supplementary material for: Effects of Vitamin D Supplementation on Lipid Profile in Adults with the Metabolic Syndrome: A Systematic Review and Meta-Analysis of Randomized Controlled Trials
Source: Nutrients. 2020 Oct 30;12(11):3352. doi: 10.3390/nu12113352 (PMC7692169; doi:10.3390/nu12113352)
Supplement: Supplementary file 1 [file nutrients-12-03352-s001.zip › Supplementary/Supplement 1- Search Strategy.docx]

**Supplement 1: Search strategy**

**Medline**

--------------------------------------------------------------------------------

1 exp Vitamin D/ (59046)

2 (Cholecalciferol* or calciol or HYDROXYCHOLECALCIFEROL* or hydroxyvitamins d or CALCIFEDIOL or 25 hydroxyvitamin d3 or 25-hydroxycholecalciferol or calcidiol or DIHYDROXYCHOLECALCIFEROL* or dihydroxyvitamins d or CALCITRIOL or 1 alpha,25 dihydroxyvitamin d3 or 1 alpha,25-dihydroxycholecalciferol or 1,25-dihydroxyvitamin d3 or ergocalciferol* or Dihydrotachysterol or 25-Hydroxyvitamin D 2).mp. (39207)

3 (vitamin adj (d or d2 or d3 or d-2 or d-3)).mp. (77923)

4 Vitamin D Deficiency/ (15801)

5 or/1-4 (90422)

6 exp Metabolic Syndrome/ (31623)

7 ((syndrome adj X) or (reaven* adj syndrome*) or (dysmetabolic adj syndrome*) or (X adj syndrome) or (Cardiovascular adj syndrome*) or Hyperinsulin* or (metabolic adj1 cardiovascular) or ((metabolism or metabolic) adj1 syndrome*) or (insulin adj1 (sensitivity or resistance))).mp. (170941)

8 or/6-7 (170941)

9 ((randomized controlled trial or controlled clinical trial).pt. or randomized.ab. or placebo.ab. or drug therapy.fs. or randomly.ab. or trial.ab. or groups.ab.) not (exp animals/ not humans.sh.) (4127916)

10 5 and 8 and 9 (854)

***************************

**Pubmed**

| **Search number** | **Query** | **Results** |
| --- | --- | --- |
| 17 | #15 and #16 | 1,028 |
| 16 | ((randomized controlled trial[pt]) OR (controlled clinical trial[pt]) OR (randomized[tiab] OR randomised[tiab]) OR (placebo[tiab]) OR (drug therapy[sh]) OR (randomly[tiab]) OR (trial[tiab]) OR (groups[tiab])) NOT (animals[mh] NOT humans[mh]) | 4,204,438 |
| 15 | #11 AND #7 | 2,648 |
| 11 | #8 or #9 or #10 | 194,557 |
| 10 | syndrome X[tw] or reaven syndrome*[tw] or dysmetabolic syndrome*[tw] or X syndrome[tw] or Cardiovascular syndrome*[tw] or metabolic cardiovascular[tw] or metabolism syndrome*[tw] or insulin sensitivit* [tw] or insulin resistan*[tw] or Hyperinsulin*[tw] | 131,392 |
| 9 | Metabolic syndrome*[tw] | 57,560 |
| 8 | metabolic syndrome | 84,120 |
| 7 | #1 or #2 or #3 or #4 or #5 or #6 | 90,577 |
| 6 | vitamin d[tw] or vitamin d2[tw] or vitamin d3[tw] or vitamin d 3[tw] or vitamin d 2[tw] | 77,299 |
| 5 | dihydroxyvitamin d[tw] or dihydroxyvitamins d[tw] or 1 alpha,25 dihydroxyvitamin d3[tw] or 1 alpha,25-dihydroxycholecalciferol[tw] or 1,25-dihydroxyvitamin d3[tw] or 25-Hydroxyvitamin D 2[tw] | 12,001 |
| 4 | 25 hydroxyvitamin d3 [tw] | 2,901 |
| 3 | Cholecalciferol*[tw] or calciol[tw] or HYDROXYCHOLECALCIFEROL*[tw] or CALCIFEDIOL[tw] or 25-hydroxycholecalciferol[tw] or calcidiol[tw] or DIHYDROXYCHOLECALCIFEROL*[tw] or CALCITRIOL[tw] or alpha,25-dihydroxycholecalciferol[tw] or ergocalciferol*[tw] or Dihydrotachysterol[tw] | 37,271 |
| 2 | vitamin D deficiency [mesh:noexp] | 15,818 |
| 1 | vitamin d | 86,073 |

**Cinahl**

| **#** | **Query** | **Results** |
| --- | --- | --- |
| S9 | S5 AND S8 | 852 |
| S8 | S6 or S7 | Display |
| S7 | TI (syndrome X or reaven* syndrome* or dysmetabolic syndrome* or X syndrome or Cardiovascular syndrome* or metabolic cardiovascular or metabolism syndrome* or metabolic syndrome* or insulin sensitivity or insulin resistance or Hyperinsulin*) or AB (syndrome X or reaven* syndrome* or dysmetabolic syndrome* or X syndrome or Cardiovascular syndrome* or metabolic cardiovascular or metabolism syndrome* or metabolic syndrome* or insulin sensitivity or insulin resistance or Hyperinsulin*) | Display |
| S6 | (MH "Metabolic Syndrome X+") | Display |
| S5 | S1 OR S2 or S3 or S4 | Display |
| S4 | TI (alpha,25-dihydroxycholecalciferol or ergocalciferol* or dihydrotachysterol or dihydroxyvitamin D or dihydroxyvitamins D or 1 alpha,25 dihydroxyvitamin d3 or 1 alpha,25-dihydroxycholecalciferol or 1,25-dihydroxyvitamin d3 or 25-hydroxyvitmain D 2) or AB (alpha,25-dihydroxycholecalciferol or ergocalciferol* or dihydrotachysterol or dihydroxyvitamin D or dihydroxyvitamins D or 1 alpha,25 dihydroxyvitamin d3 or 1 alpha,25-dihydroxycholecalciferol or 1,25-dihydroxyvitamin d3 or 25-hydroxyvitmain D 2) | Display |
| S3 | TI (vitamin d or vitamin d2 or vitamin d3 or vitamin d 3 or vitamin d 2) or AB (vitamin d or vitamin d2 or vitamin d3 or vitamin d 3 or vitamin d 2) | Display |
| S2 | TI (Cholecalciferol* or calciol or HYDROXYCHOLECALCIFEROL* or hydroxyvitamin* d or CALCIFEDIOL or 25 hydroxyvitamin d3 or 25-hydroxycholecalciferol* or calcidiol or DIHYDROXYCHOLECALCIFEROL* or dihydroxyvitamin* d or CALCITRIOL or 1 alpha,25 dihydroxyvitamin d3 or 1 alpha,25-dihydroxycholecalciferol or 1,25-dihydroxyvitamin d3 or ergocalciferol* or Dihydrotachysterol* or 25-Hydroxyvitamin D 2) or AB (Cholecalciferol* or calciol or HYDROXYCHOLECALCIFEROL* or hydroxyvitamin* d or CALCIFEDIOL or 25 hydroxyvitamin d3 or 25-hydroxycholecalciferol* or calcidiol or DIHYDROXYCHOLECALCIFEROL* or dihydroxyvitamin* d or CALCITRIOL or 1 alpha,25 dihydroxyvitamin d3 or 1 alpha,25-dihydroxycholecalciferol or 1,25-dihydroxyvitamin d3 or ergocalciferol* or Dihydrotachysterol* or 25-Hydroxyvitamin D 2) | Display |
| S1 | MH “Vitamin D+” OR MH “Vitamin D deficiency+” | Display |

**Embase**

--------------------------------------------------------------------------------

1 exp vitamin D/ (145277)

2 exp vitamin D deficiency/ (30164)

3 (Cholecalciferol* or calciol or HYDROXYCHOLECALCIFEROL* or hydroxyvitamins d or CALCIFEDIOL or 25 hydroxyvitamin d3 or 25-hydroxycholecalciferol or calcidiol or DIHYDROXYCHOLECALCIFEROL* or dihydroxyvitamins d or CALCITRIOL or 1 alpha,25 dihydroxyvitamin d3 or 1 alpha,25-dihydroxycholecalciferol or 1,25-dihydroxyvitamin d3 or ergocalciferol* or Dihydrotachysterol or 25-Hydroxyvitamin D 2).mp. (53512)

4 (vitamin adj (d or d2 or d3 or d-2 or d-3)).mp. (135546)

5 or/1-4 (168091)

6 exp metabolic Syndrome/ (83594)

7 ((syndrome adj X) or (reaven* adj syndrome*) or (dysmetabolic adj syndrome*) or (X adj syndrome) or (Cardiovascular adj syndrome*) or (metabolic adj1 cardiovascular) or (metabolic adj1 syndrome*) or (metabolism adj1 syndrome*) or Hyperinsulin* or (insulin adj1 (sensitivity or resistance))).mp. (283771)

8 or/6-7 (283771)

9 5 and 8 (5299)

10 crossover-procedure/ or double-blind procedure/ or randomized controlled trial/ or single-blind procedure/ or (random* or factorial* or crossover* or cross over* or placebo* or (doubl* adj blind*) or (singl* adj blind*) or assign* or allocat* or volunteer*).tw. (2382891)

11 9 and 10 (941)

***************************

**The Cochrane Library**

ID Search Hits

#1 MeSH descriptor: [Vitamin D] explode all trees 5310

#2 MeSH descriptor: [Vitamin D Deficiency] explode all trees 1420

#3 (Cholecalciferol* or calciol or HYDROXYCHOLECALCIFEROL* or CALCIFEDIOL):ti,ab,kw 3445

#4 (calcidiol or DIHYDROXYCHOLECALCIFEROL* or CALCITRIOL):ti,ab,kw 1930

#5 (ergocalciferol* or Dihydrotachysterol or dihydroxyvitamin d or dihydroxyvitamins d or 1 alpha,25 dihydroxyvitamin d3):ti,ab,kw 2168

#6 (vitamin d):ti,ab,kw 13817

#7 (vitamin d2 or vitamin d 3):ti,ab,kw 7916

#8 #1 or #2 or #3 or #4 or #5 or #6 or #7 15467

#9 MeSH descriptor: [Metabolic Syndrome] explode all trees 1713

#10 (metabolic syndrome):ti,ab,kw 8835

#11 (syndrome near/1 metabolic or reaven* syndrome* or dysmetabolic near/1 syndrome* or X near/1 syndrome or Cardiovascular near/1 syndrome* or metabolic near/1 cardiovascular or metabolism near/1 syndrome* or insulin near/1 sensitivity or insulin near/1 resistance or hyperinsulin*):ti,ab,kw 22436

#12 #9 or #10 or #11 23986

#13 #8 AND #12 900

**Clinicaltrials.gov**

Vitamin D | Metabolic Syndrome | Adult

**WHO International Clinical Trials Registry Platform**

Vitamin D AND metabolic syndrome
