# Supplementary material for: Effects of Vitamin D Supplementation on Lipid Profile in Adults with the Metabolic Syndrome: A Systematic Review and Meta-Analysis of Randomized Controlled Trials
Source: Nutrients. 2020 Oct 30;12(11):3352. doi: 10.3390/nu12113352 (PMC7692169; doi:10.3390/nu12113352)
Supplement: Supplementary file 1 [file nutrients-12-03352-s001.zip › Supplementary/Supplement 2- GRADEing of the MA.docx]

**Supplement 2: GRADEing of the Meta-analysis**

**Table 1: Quality of evidence of included RCTs investigating the effect of low-dose vitamin D supplementation compared with no supplementation on dyslipidemia in adults with the metabolic syndrome**

| **Certainty assessment** | | | | | | **№ of patients** | | **Effect** | **Certainty** |
| --- | --- | --- | --- | --- | --- | --- | --- | --- | --- |
| **№ of studies** | **Study design** | **Risk of bias** | **Inconsistency** | **Indirectness** | **Imprecision** | **Vitamin D supplementation** | **No vitamin D supplementation** | **Absolute (95% CI)** |  |
| **LDL-C** | | | | | | | | | |
| 3 | Randomized trials | Serious ^a^ | Very serious ^b^ | Not serious | Very serious ^c^ | 77 | 78 | MD **20.08 lower** (49.7 lower to 9.55 higher) | ⨁◯◯◯ VERY LOW |
| **HDL-C** | | | | | | | | | |
| 3 | Randomized trials | Serious ^a^ | Not serious | Not serious | Very serious ^c^ | 77 | 78 | MD **0.11 higher** (3.04 lower to 3.26 higher) | ⨁◯◯◯ VERY LOW |
| **TC** | | | | | | | | | |
| 3 | Randomized trials | Serious ^a^ | Serious ^d^ | Not serious | Serious ^e^ | 77 | 78 | MD **16.76 lower** (36.47 lower to 2.94 higher) | ⨁◯◯◯ VERY LOW |
| **Triglycerides** | | | | | | | | | |
| 2 | Randomized trials | Serious ^f^ | Very serious ^b^ | Not serious | Serious ^g^ | 52 | 53 | MD **30.67 higher** (4.89 higher to 56.45 higher) | ⨁◯◯◯ VERY LOW |

*LDL-C: Low-density lipoprotein cholesterol; HDL-C: High-density lipoprotein cholesterol; TC: Total cholesterol; CI: Confidence interval; MD: Mean difference*

a. Downgraded by one level due to serious risk of bias. Unclear sequence generation, allocation concealment and blinding of participants and personnel.

b. Downgraded by two levels due to very serious inconsistency. I^2^=86%

c. Downgraded by two levels due to very serious imprecision. Confidence interval includes both values suggesting benefit and values suggesting harm. Low number of participants.

d. Downgraded by one level due to serious inconsistency. I^2^=62%

e. Downgraded by one level due to serious imprecision. Confidence interval includes both values suggesting benefit and no effect. Low number of participants.

f. Downgraded by one level due to serious risk of bias. Unclear sequence generation and allocation concealment.

g. Downgraded by one level due to serious imprecision. Confidence interval includes both values suggesting no effect and harm. Low number of participants.

**Table 2: Quality of evidence of included RCTs investigating the effect of high-dose of vitamin D supplementation compared with no supplementation on dyslipidemia in adults with the metabolic syndrome**

| **Certainty assessment** | | | | | | **№ of patients** | | **Effect** | **Certainty** |
| --- | --- | --- | --- | --- | --- | --- | --- | --- | --- |
| **№ of studies** | **Study design** | **Risk of bias** | **Inconsistency** | **Indirectness** | **Imprecision** | **Vitamin D supplementation** | **No vitamin D supplementation** | **Absolute (95% CI)** |  |
| **LDL-C** | | | | | | | | | |
| 2 | Randomized trials | Not serious ^a^ | Not serious | Not serious | Very serious ^b^ | 63 | 64 | MD **1.12 lower** (10.94 lower to 8.7 higher) | ⨁⨁◯◯ LOW |
| **HDL-C** | | | | | | | | | |
| 2 | Randomized trials | Not serious ^a^ | Not serious | Not serious | Very serious ^b^ | 63 | 64 | MD **0.2 lower** (3.28 lower to 2.88 higher) | ⨁⨁◯◯ LOW |
| **TC** | | | | | | | | | |
| 2 | Randomized trials | Not serious ^a^ | Not serious | Not serious | Very serious ^b^ | 63 | 64 | MD **6.07 higher** (5.97 lower to 18.12 higher) | ⨁⨁◯◯ LOW |
| **Triglycerides** | | | | | | | | | |
| 2 | Randomized trials | Not serious ^a^ | Serious ^c^ | Not serious | Serious ^d^ | 63 | 64 | MD **27.33 higher** (2.06 higher to 52.59 higher) | ⨁⨁◯◯ LOW |

*LDL-C: Low-density lipoprotein cholesterol; HDL-C: High-density lipoprotein cholesterol; TC: Total cholesterol; CI: Confidence interval; MD: Mean difference*

a. Concern about unclear sequence generation and allocation concealment in one study.

b. Downgraded by two levels due to very serious imprecision. Confidence interval includes both values suggesting benefit and values suggesting harm. Low number of participants.

c. Downgraded by one level due to serious inconsistency. I^2^=51%

d. Downgraded by one level due to serious imprecision. Confidence interval includes both values suggesting no effect and values suggesting benefit. Low number of participants.
