# Supplementary material for: Effects of Vitamin D Supplementation on Lipid Profile in Adults with the Metabolic Syndrome: A Systematic Review and Meta-Analysis of Randomized Controlled Trials
Source: Nutrients. 2020 Oct 30;12(11):3352. doi: 10.3390/nu12113352 (PMC7692169; doi:10.3390/nu12113352)
Supplement: Supplementary file 1 [file nutrients-12-03352-s001.zip › Supplementary/Supplement 3- Sensitivity analyses.docx]

**Supplement 3: Sensitivity analyses**

Since heterogeneity was high for the 3 outcomes (LDL-C, HDL-C and TC). Sensitivity analyses were performed by excluding Farag et al. (2019), since the baseline characteristics (baseline levels) in the two randomized arm of this study were not similar. Heterogeneity dropped to none, the 95% confidence interval was more precise for LDL-C, compared with the base-case analysis, while that of HDL-C did not substantively change.


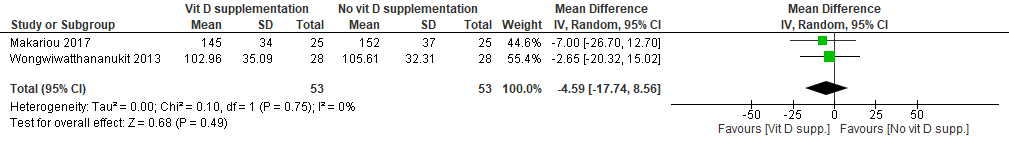


**Figure 1a. Forest plot of sensitivity analysis of mean differences in LDL-C (in mg/dL) between subjects receiving low-dose vitamin D supplementation compared with those not receiving vitamin D supplementation (excluding Farag, 2019 in which the baseline values were not similar in the randomized arms)**


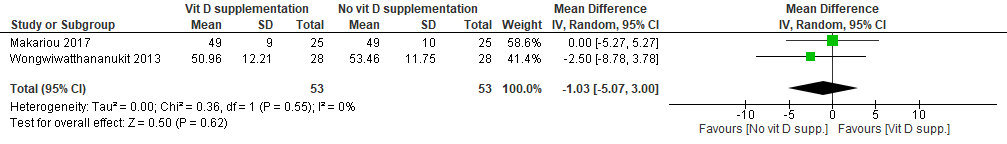


**Figure 1b. Forest plot of sensitivity analysis of mean differences in HDL-C (in mg/dL) between subjects receiving low-dose vitamin D supplementation compared with those not receiving vitamin D supplementation (excluding Farag, 2019 in which the baseline values were not similar in the randomized arms)**


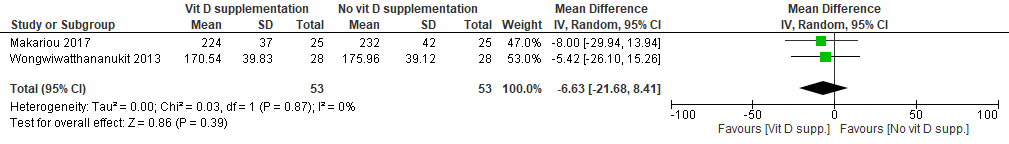


**Figure 1c. Forest plot of sensitivity analysis of mean differences in TC (in mg/dL) between subjects receiving low-dose vitamin D supplementation compared with those not receiving vitamin D supplementation (excluding Farag, 2019 in which the baseline values were not similar in the randomized arms)**

**Figure 1. Meta-analysis of the sensitivity analyses of the effects of low-dose vitamin D supplementation on LDL-C, HDL-C and TC. Mean differences for each study are represented by squares, and 95% Confidence Intervals are represented by the lines through the squares. The pooled mean differences are represented by diamonds. Between-study heterogeneity was assessed with the use of the I^2^ statistic.**

*LDL-C: Low-density Lipoprotein Cholesterol; HDL-C: High-density Lipoprotein Cholesterol; TC: Total Cholesterol.*
